# Adoptive cellular immunotherapy for solid neoplasms beyond CAR-T

**DOI:** 10.1186/s12943-023-01735-9

**Published:** 2023-02-07

**Authors:** Qiaofei Liu, Jiayi Li, Huaijin Zheng, Sen Yang, Yuze Hua, Nan Huang, Jorg Kleeff, Quan Liao, Wenming Wu

**Affiliations:** 1 Department of General Surgery, State Key Laboratory of Complex Severe and Rare Diseases, Peking Union Medical College Hospital, Peking Union Medical College, Chinese Academy of Medical Sciences, No.1 Shuai Fu Yuan, Dongcheng District, Beijing, 100730 China; 2 Department of Visceral, Vascular and Endocrine Surgery, Martin-Luther-University Halle-Wittenberg, 06120 Halle (Saale), Germany

**Keywords:** Adoptive cell therapy, Immune checkpoint, Chimeric antigen receptor, TCR, Natural killer cell, Macrophage

## Abstract

In recent decades, immune checkpoint blockade and chimeric antigen receptor T cell (CAR-T) therapy are two milestone achievements in clinical immunotherapy. However, both show limited efficacies in most solid neoplasms, which necessitates the exploration of new immunotherapeutic modalities. The failure of CAR-T and immune checkpoint blockade in several solid neoplasms is attributed to multiple factors, including low antigenicity of tumor cells, low infiltration of effector T cells, and diverse mechanisms of immunosuppression in the tumor microenvironment. New adoptive cell therapies have been attempted for solid neoplasms, including TCR-T, CAR-natural killer cells (CAR-NK), and CAR-macrophages (CAR-M). Compared to CAR-T, these new adoptive cell therapies have certain advantages in treating solid neoplasms. In this review, we summarized the 40-year evolution of adoptive cell therapies, then focused on the advances of TCR-T, CAR-NK, and CAR-M in solid neoplasms and discussed their potential clinical applications.

## Introduction

Patients with primary or acquired immune deficiency suffer a higher risk of various malignancies at an earlier age than the immunocompetent population [1, 2]. A functional immune system is essential to prevent tumorigenesis by recognizing, targeting, killing, and scavenging the nascent transformed cells [3]. However, the accumulations of genetic mutations in the transformed cells result in an enhanced capacity to escape from immune surveillance. Further, the immune cells can be re-educated to promote progression and metastasis [4]. Therefore, reconstructing or armoring the immune system offers a potential clinical treatment for various cancers.

At the end of the last century, adoptive cell therapies (ACT) using autologous peripheral lymphocytes or tumor-infiltrating lymphocytes (TILs) after stimulation and expansion by lymphokines have been widely attempted in the clinical setting. However, significant clinical effects were only achieved in certain tumors (mainly in melanoma) [5–7]. This first-generation ACT lacked specific targeting and killing abilities. Therefore, specific targeting ACT has emerged since then. Taking advantage of the specific binding capacity of the extracellular single chain variable region of an immunoglobulin and the activation capacity of the intracellular region of the T cell receptor (TCR), artificial chimeric antigen receptors (CAR) were constructed and then transduced into autologous cytotoxic T lymphocytes to build CAR-T [8, 9]. Although CAR-T treatments by targeting CD19, CD20, and B-cell maturation antigen (BCMA) have been successful in hematopoietic neoplasm (B cell leukemia and lymphoma and multiple myeloma), leading to five Food and Drug Admiration (FDA) approved therapies since 2017, their efficacies in solid neoplasms have been disappointing. As of now, no CAR-T product has been approved for solid neoplasms [10–13]. Many factors contribute to CAR-T’s limited efficacy in solid neoplasms. First, few solid tumors produce tumor-specific antigens as targets for CAR-T. Although CAR-Ts targeting HER2, mesothelin, MUC1, and PSMA have been applied in the preliminary trials of breast, pancreatic, and prostate cancer, they showed limited specific killing abilities [14]. Second, in most solid tumors, T cells are a minor proportion of the immune cell infiltration [15]. Third, the interaction between cancer cells and the tumor microenvironment (TME) components builds an immunosuppressive environment to dampen the killing abilities of T cells [4, 16–18]. As another milestone achievement of clinical immunotherapy, immune checkpoint blockade (ICB, including anti-PD1/anti-PDL1 and CTL4) aiming to re-activate the dampened cytotoxic T cells is clinically successful in certain advanced-stage cancers (melanoma, non-small cell lung cancer, hepatocellular carcinoma, Hodgkin’s lymphoma), facilitating the FDA approval of more than five checkpoint inhibitors in the US [19–22]. The potential synergistic effects of CAR-T and ICB have been demonstrated in the pre-clinical setting, and several clinical trials are going on [23, 24]. Another primary concern of CAR-T is its high incidence of serious adverse effects that are potentially life-threatening, including graft versus host diseases (GVHD), cytokine release syndrome (CRS), and neurotoxicity [25].

Considering the limitations of CAR-T, some new ideas for ACTs have been emerging. One is an alternative cell of origin to construct armed autologous or allogenous immune cells with the CARs; the other is to screen and identify HLA-restricted tumor-specific targeting-TCR and then build genetically engineered autologous TCR-T cells. NK cells and macrophages are the major players of innate immune cells in solid tumors [15]. NK cells share a similar cytotoxic function with CD8-positive cells by releasing perforin and granzyme. CAR-NK cells have antigen-independent and antigen-dependent tumor-killing capacities simultaneously [26]. Macrophages are the predominant immune cell populations in the majority of solid tumors and they are highly diverse and show dynamic changes in phenotype and functions between the M1 and M2 subtypes by stimuli from the TME [16]. CAR-macrophage (CAR-M) has certain potential advantages over CAR-T, including high intra-tumoral migration capacity, antigen-dependent/independent phagocytosis, enhanced antigen-presenting capacity, and remodeling the immunosuppressive microenvironment [27]. Other cells, such as umbilical cord blood (UCB) and induced pluripotent stem cells (iPSCs), are under research to construct CAR-NKs or CAR-Ms induced and stimulated by interleukins and cytokines [26, 28, 29]. CAR-T recognizes the surface antigens by binding to the scFv region of an immunoglobulin; however, TCR-T, armed with HLA-restricted tumor-specific TCR, exerts function only when binding to intracellular tumor-specific antigens which are presented by specific HLA molecules. Several studies reported that CAR-M, CAR-NK, and TCT-R therapy have a lower risk of CRS and neurotoxicity [30].

**Fig. 1:**
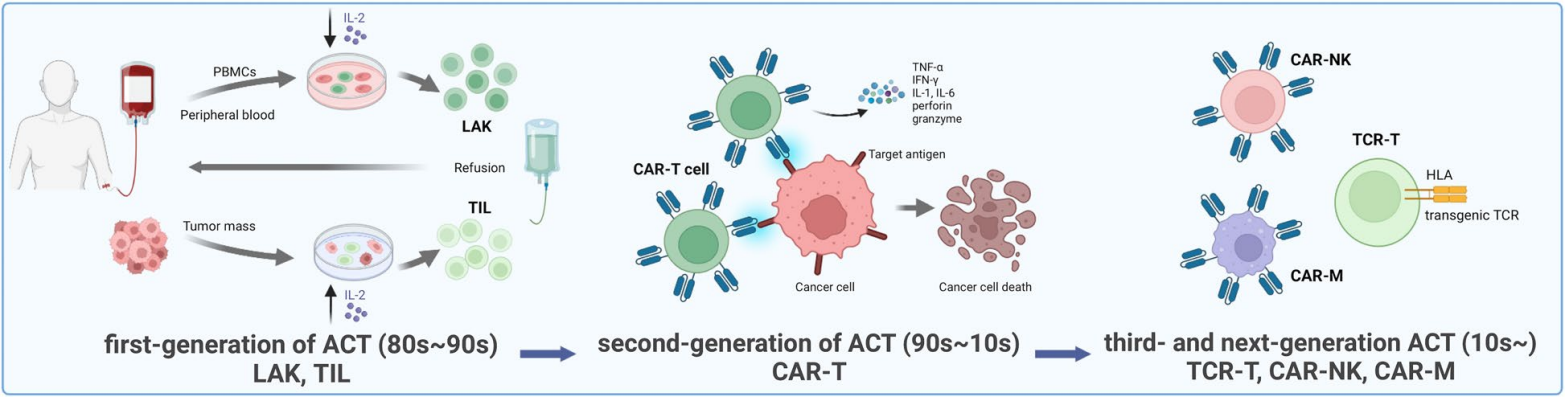
Evolution of ACTs during the last four decades

The mechanisms, evolutions, advances, clinical applications, and toxicity management of CAR and CAR-T have been comprehensively reviewed (The brief 40-year history of ACT is depicted in Fig. 1). They are out of the scope of our review [31–34]. Herein, we mainly focus on the development and advances of CAR-M, CAR-NK, and TCR-T and their potential clinical applications.

## A brief 40-year history of ACT

### The era of the first-generation ACT

In 1982, Rosenberg and colleagues established a novel in vitro culture system to activate and expand autologous peripheral lymphocytes, consisting of NK, T cells, NKT cells, and monocytes from peripheral blood, by -besides others- adding interleukin-2 (IL-2). These cultured lymphocytes showed more assertive and distinctive killing capacities over NK cells and cytotoxic T lymphocytes (CTLs); therefore, these cells were called lymphokine-activated killer cells (LAK) [35]. Later, it was reported that the transfusion of TIL stimulated by IL-2 showed more robust killing capacities over LAK from peripheral blood lymphocytes [5]. However, neither TIL nor LAK alone showed substantial effects in animal models and preliminary clinical trials [6]. Only in combination with IL-2 could they show effects. Although the availability of recombinant IL-2 tremendously lowered the cost of TIL and LAK treatment, which led to a surge of clinical attempts in varieties of the advanced stage of solid tumors from the 1990s to 2000s, this first generation of ACT did not have a specific targeting-killing capacity which would lead to unpredictable clinical outcome. In addition, the high incidence of serious adverse events after infusion of a high dose of IL-2 further limits its broad clinical applicability [36–39].

### Era of CAR-T

**Fig. 2:**
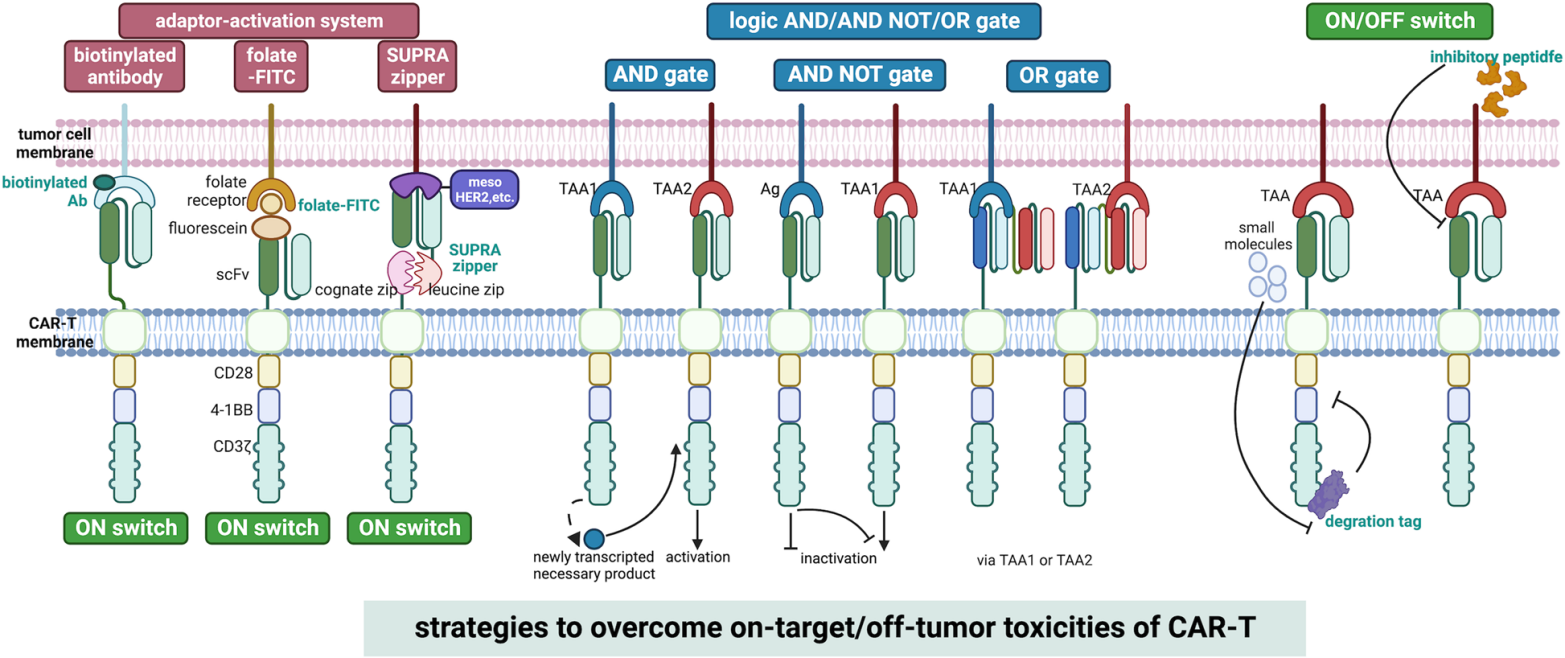
Mechanisms of logical controls of on-target/off-tumor of CAR-T

**Fig. 3:**
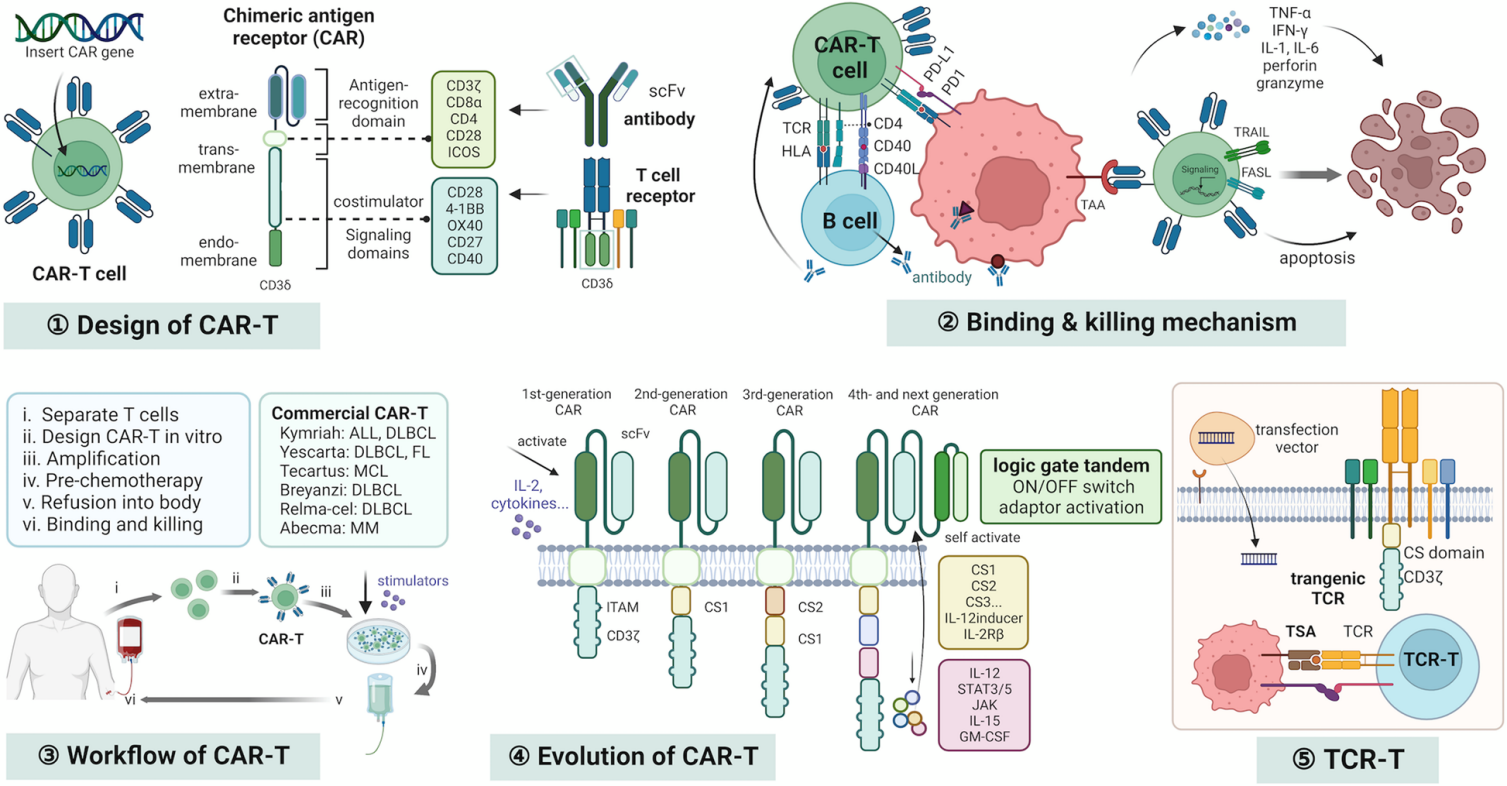
Mechanisms and development of CAR-T and TCR-T

The rapid progression of gene engineering from the 1980s established new approaches in the field of ACT. The first CAR containing the variable region of an antibody and constant region of T cell receptor was described in 1987 by Yoshihisa Kuwana [8]. In their study, VL-Cβ and VH-Cα gene, and VL-Cα and VH-Cβ gene, were genetically inserted into an expression vector and transferred in EL4 cells. This CAR could be activated by a specific antigen. In 1991, Arthur et al. constructed a CAR with the combination of the intracellular signaling domain of CD3ζ, which represented the final structure of the first-generation CAR consisting of CD3ζ intracellular domains and single-chain fraction variable (scFv) domain of an immunoglobulin [40]. However, the clinical trials of the first generation of CAR-T showed dismal results. In early 2000, the importance of co-stimulators was recognized, and the domains of the co-stimulators (CD28, 4-1BB) were inserted into the structure of CARs, leading to the second generation of CAR. Positive results in a series of clinical trials of these second-generation CAR-T targeting CD19 and CD20 in hematopoietic malignancies validated the first FDA approval of CAR-T (Tisagenlecleucel, Kymriah, and Novartis) in 2017 [13, 41–43]. To improve its efficacy, the insertion of multiple domains from co-stimulators (CD28, CD27, OX40, 4-1BB, etc.) helped construct the third-generation of CARs, as well as the addition of immunomodulatory factors, including cytokines, chemokines, growth factors, interferon (IFN), and tumor necrosis factor (TNF) to build the fourth-generation of CARs. To guarantee the safety of CARs, logic-gate combinations, such as the ON/OFF switch and adaptor-mediation of CAR-T activation were proposed. The logic-gate includes AND-gate, AND/NOT-gate, and OR-gate. AND-gate requires the co-existence of multiple tumor antigens to activate CAR-T reducing the risk of off-target recognition. AND/NOT-gate will prevent activation of CAR-T when a normal antigen is recognized. OR-gate will activate a CAR-T through any of the tandem scFvs. The stimuli or small molecules are designed as the controller to turn the switch on/off. Adaptor-activation systems only allow CAR-T activation when the adaptors are recognized, including biotinylated Ab, Folate-FITC, SUPRA (slit, universal and programmable) Zipper system, and others. The SUPAR Zipper system of CAR consists of a switch-molecule with a leucine zipper linked to TAA-binding scFv and a paired cognate leucine zipper containing a universal receptor linked to the intracellular signal domain [44]. The details of the advances in the design of CARs have been comprehensively discussed elsewhere [34, 45, 46]. The mechanisms of logical controls of on-target/off-tumor were depicted in Fig. 2. Although CAR-T has been won great success in hematopoietic malignancies, its role in most solid carcinomas remains limited. Missing targetable surface tumor antigens, a low number of T cells in the tumor microenvironment, and immunosuppression contribute to the low efficacy (Fig. 3).

### Era beyond CAR-T

In the 2010s, two new directions of ACT design emerged: the genetically modified TCR-T and the new origin effector immune cells armed by CARs. Autogenous or allogenous T cells can now express TCRs recognizing multiple combinations of specific peptides and human leukocyte antigens (HLA) through progress in TCR isolating, sequencing, and genetic engineering techniques. Unlike CAR, which recognizes the antigens on the cell surface, TCR only recognizes the intracellular antigens presented by specific HLA molecules. Few solid tumors have specific surface antigens. In contrast, gene mutations potentially produce altered intracellular proteins, which may be presented by HLA molecules [47]. In 2016, Tran et al. reported a successful clinical case of HLA-C*08:02 restricted TCR-T targeting KRAS G12D isolated from TIL in metastatic colorectal cancer [48]. Further, a successful case in metastatic pancreatic cancer by using trans-genetically inserted autologous HLA-C*08:02 restricted TCR-T targeting KRASG12D was reported [49]. However, the incidence of the HLA-C*08:02 allele is lower than 10% in the population.

Macrophages and NK cells are the primary innate cell populations in TME with different cytotoxic effects compared to T cells. Macrophages perform multiple functions, including phagocytosis, immunomodulation, TME remodeling, and antigen-presenting. CAR-M-targeting-HER2 has specific phagocytosis capacity in vitro and murine breast cancer models whose immunosuppressive microenvironment could be improved in a HER2-dependent way [27, 50]. CAR-NKs potentially eliminate tumor cells via NCRs, NKG2D, DNAM-1 (CD226) and certain activating KIRs (KIR2DS1, KIR2DS4, and KIR2DL4) independent of CAR, as well as through CD16-mediated antibody-dependent cell-mediated cytotoxicity (ADCC) [51].

## TCR-T

### Mechanisms of activation and regulation of TCR

T lymphocytes are the primary adaptive immune effector cells. They recognize HLA-presented antigens by TCR with the help of CD3, CD4, CD8, and co-stimulation factors [52]. Two different protein chains compose a TCR heterodimer. 95% of T cells (αβT) have TCR of α and β chains, while TCR consists of γ and δ chains in 5% of γδT cells. A transmembrane region and a short cytoplasmic tail follow an extracellular constant region, whereas a variable region binds peptides and HLAs. TCR cannot mediate signal transduction itself via its short cytoplasmic tail but requires CD3 for signal transduction [53]. Two chains of TCR and six CD3 adaptor proteins construct an octameric complex (TCR αβ - CD3εγ - CD3εδ - CD3ζζ) in the plasma membrane. The signaling motifs of CD3 can be phosphorylated in the event of TCR-HLA-antigen binding. The tyrosine residues are present in a specific amino acid sequence of Yxx(L/I)× 6-8Yxx(L/I), common in activator receptors of the non-catalytic tyrosine-phosphorylated receptor (NTR) family and referred as an immunoreceptor tyrosine-based activation motif (ITAM). A TCR complex contains 10 ITAMs in total (CD3δ, CD3γ, and CD3ε have a single ITAM, respectively, and CD3ζ has three ITAMs) [54]. The tyrosine residues of the ITAMs will be phosphorylated when the TCR binds to HLA-peptides, which is mediated by the Src kinase (Lck) anchored to the plasma membrane by the co-receptor CD4 (Th cells) or CD8 (cytotoxic T cells). The phosphatase CD45 (the common leukocyte antigen) in the membrane can remove de-phosphorylate tyrosine residues of Lck and inhibit signal initiation [55]. Phosphorylated ITAMs can recruit protein tyrosine kinase ZAP70 by binding to the phosphorylated tyrosine residues in its SH2 domain. Subsequently, ZAP70 is phosphorylated by Lck and activated, and can phosphorylate multiple tyrosine residues of the transmembrane protein LAT, a scaffold protein without any catalytic activity but provides binding sites for signaling molecules via phosphorylated tyrosine residues. LAT cooperates with another folding protein to form a cooperative signalosome as a platform to recruit downstream signaling molecules, including phospholipase Cγ1 (PLCγ1), SOS, Itk, Vav, Nck1, and Fyb. Then, diacyl glycerol (DAG) and inositol 1,4,5-trisphosphate (IP3), the second messengers, are generated with the help of the co-stimulation factors (CD28, CD40L, ICOS). They amplify the TCR signal and activate transcription factors, including NFAT, NF-κb, and AP1 [56, 57]. The inhibitory stimulation factors, CTLA-4 and PD1, attenuate the activation of T cells [20, 58, 59]. The intracellular domain of CTLA4 contains one motif binding to PP2A, PI3K, and SHP-2 and another to SH3. It is similar to that of CD28 but has no intrinsic catalytic activity. CTLA-4 can dephosphorylate TCR-proximal signaling proteins ZAP70 and LAT directly via SHP-2 and PP2A, as well as inhibiting the signaling indirectly by competing with CD28 for CD80/86 binding [60]. The intracellular tail of PD-1 contains two phosphorylation sites located in an immunoreceptor tyrosine-based inhibitory motif and an immunoreceptor tyrosine-based switch motif, consistent with the binding of SHP-1 and SHP-2 to the cytoplasmic tail of PD-1 upon ligand binding. Also, PD-1 can inhibit T cell receptor activation by up-regulating E3-ubiquitin ligases CBL-b and c-CBL. PD-1 expression on activated T cells, B cells, and macrophages indicates that it has a broader immune inhibitory role than CTLA4 [61, 62].

### CAR-T vs. TCR-T

CAR is an artificial combined TCR, combining the advantages of immunoglobulin, co-stimulation factors, and CD3. The extracellular scFv region from immunoglobulin in a CAR has a high affinity to specific surface antigens without the assistance of HLA molecules. The intracellular region contains single or dual co-stimulatory domains and a CD3ζ motif in CAR that can activate CAR-T cells. CAR-T can be activated upon binding a specific surface antigen to exert killing functions [14]. Since CAR can be activated without CD3 or co-stimulation factors, it can be genetically transduced into other immune cells, including macrophages and NK cells [26, 27]. However, transmembrane peptides constitute only an estimated 14–26% of the proteome making CAR-targetable antigens limited. CAR can also target glycoproteins and glycolipids as extended antigens, while TCR only recognizes the HLA-restricted peptides from intracellular components with the assistance of CD3, CD4, CD8, co-stimulation factors and so on. Therefore, TCR could hardly be genetically inserted into other immune cells except T lymphocytes [63]. With more than 20,000 HLA alleles identified, HLA-encoding genes are the most polymorphic in the genome. It is important to screen and identify tumor-specific peptide-HLA complexes as the TCR target. The HLA-A*02:01 allele present in about 47.8 and 16.8% of the Caucasian and African American population is the most commonly tested loci to date [64, 65]. Indeed, the expressions of target antigens on normal cells would cause on-target/off-tumor toxicity. CARs could mediate supraphysiologic T-cell activation, leading to enhanced cytokine release. Furthermore, the CRS in CAR-Ts is more common than in TCR-Ts. Tumor antigens in cancer cells are predominantly intracellular; therefore, TCR-T may prevail over CAR-T in targeting cancer cells and minimizing toxicity [66, 67].

### Tumor antigens for TCR-T in solid tumors

Tumor antigens include tumor-specific antigens (TSA) and tumor-associated antigens (TAA). TSAs are expressed in cancer cells but not in normal cells due to gene mutations or viral infections. Gene mutations can lead to various peptides with different immunogenicity than their normal forms. These new peptides, called neoantigens, can be processed and presented by the HLA molecule to the TCR to activate T lymphocytes. Since genomic instability occurs in most cancers, these neoantigens are the best candidates for TCR-T therapy. Some neoantigens may be produced in various cancers, are restricted to a common HLA molecule, and are called “public neoantigens”. Currently, the KRAS G12D/G12V mutations, which are found in over 70% of pancreatic adenocarcinoma and in over 30% of colorectal carcinoma, and PIK3CA H2047L, which is present in 5% of metastatic breast cancer, have been demonstrated to be effective HLA-restricted public neoantigens for TCR-T treatment [68–70]. However, their HLA-restricted alleles are present only in less than 10% of a minor population. Virus infections are the direct cause of tumorigenesis in several cancers, including human papillomavirus (HPV) in cervical carcinoma, hepatitis B/C virus (HBV/HCV) in hepatocellular cancer, and Epstein-Barr virus (EBV) in nasopharyngeal carcinoma. Viral oncogenes can serve as TCR-T targets since they are often expressed in exclusive virus-driven cancers rather than normal tissues. The viral antigens E6 and E7 from HPV and LMP1 and LMP2 from EBV are discovered in various solid tumors and tested in several preliminary clinical trials of TCR-T treatment [71–73].

TAAs can be classified into differentiation antigens and overexpressed antigens. Overexpressed antigens have a higher expression level in cancer cells than in normal cells. Targeting overexpressed antigens would pose a high risk for on-target/off-tumor toxicity. Wilm’s Tumor Antigen (WT1) is a kind of TAA with a 50–100-fold increased expression in leukemic cells. Several preliminary clinical TCR-T trials targeting WT1 in leukemia were conducted, and the objective response rate (ORR) ranged from 0 to 40% [74, 75]. Cancer cells, as well as the original tissue cells, express differentiation antigens. Theoretically, differentiation antigens should be tolerated by the immune system during embryo development, whereas spontaneous T-cell responses to differentiation antigens in melanoma. MART-1 and gp100 are the main differentiation peptides recognized by T cells in both cancer cells and normal melanocytes. The mechanism of incomplete tolerance against these melanocytic antigens is unclear. Several TCR-T clinical trials targeted differentiation antigens of melanoma whose on-tumor/off-target adverse effects are serious [76, 77].

Cancer germline antigens (CGAs) are expressed during the maturation of germline cells. Some CGAs (MAGE-A1, MAGE-A3, MAGE-A4, NY-ESO-1, PRAME, CT83, and SSX2) are expressed in solid neoplasm, including melanoma, liver cancer, lung cancer, bladder cancer, and neuroblastoma [78]. Since germline cells lack HLA-I molecules and fail to be recognized by CTLs, CGAs are ideal targets for TCR-T. Several preliminary clinical trials of TCR-T against CGAs have been reported (Fig. 2) [79, 80].

## Optimizing TCR-T treatment

### Screening and identifying HLA-restricted-tumor-antigens in solid tumors

High-output platforms to screen and identify HLA-restricted- tumor-antigens for TCR recognition are the prerequisite for effective TCR-T treatment [81]. To generate antigen-specific T lymphocytes, peripheral T lymphocytes can be stimulated with tumor antigens (exogenous peptides or cDNA/RNA delivery) presented by antigen-presenting cells (APCs) or artificial APCs (aAPC). One common aAPC system uses the HLA-A, B, and DR-negative myelogenous leukemia cell line K562. This aAPC cell line works after stable transduction of various HLA alleles and costimulatory molecules. In addition, cell-free aAPC systems have also been attempted by conjugation HLA and costimulatory molecules onto beads and nanoparticles [82]. Further, antigen-specific T lymphocytes can be isolated from TILs. Compared to peripheral T lymphocytes, TILs are often enriched in more TCR-specific clones. In addition, HLA-transgenic mice were also used for tumor-specific TCR screening. When tumor antigens immunize human HLA-transgenic mice, antigen-specific T cells accumulate in the lymph nodes and spleen. These tumor-specific T cells provide a TCR repertoire to further screen and identify tumor-specific TCR sequences [83, 84]. Single-cell RNA sequencing (scRNAseq) is a rapidly advancing technology. It can assess gene expression and transcripts at the single T cell level. The α and β chains of tumor-specific TCRs were successfully identified by analyzing the expressions of effector cytokines, including TNF-α, IFN-γ, and IL-2 in T cells [81, 85, 86].

### γδTCR-T treatment in solid tumors

Approximately 10% of the CD8 + T-cells express TCRs composed of γ- and δ-chains (γδT-cells). γδT-cells are different from αβT-cells in recognizing antigens, activating, and producing an antigen-specific repertoire. The γδ T cells do not require antigen processing and HLA-restricted peptide epitopes and also recognize lipid antigens [87, 88]. Non-HLA restricted antigen recognition and abundant cytokine secretion, TNF-α and IFN-γ included, suggests that γδ T cells can be as potentially effective as TCR-T treatment. However, there has been no γδ TCR-T treatment available in a clinical study.

### Synergistic strategies for TCR-T treatment in solid tumors

The loss of function mutations of HLA molecules and specific antigens are the main mechanisms of immune escape of cancer cells from T cell toxicity leading to resistance to ACT [48, 49]. Considering the different activation mechanisms of TCR-T and CAR-T, combination these two modalities may lead to synergistic effects. This combinational strategy could recognize surface antigen and cellular antigen with or without HLA restriction. In the clinical setting, TCR-T and CAR-T treatment processing is similar, including autogenous peripheral or tumor-infiltrating lymphocytes isolation, in vitro culture expansion, genetical transduction of TCR or CAR, lymphodepletion before transfusion, and transfusion of TCR-T or CAR-T with IL-2 [30, 89]. Further clinical exploration could solve the potential toxicity in certain tumors with definitive targets for combined CAR-T and TCR-T treatments. CAR-T is also capable of targeting stromal cells to block their pro-tumoral actions. A subset of TAMs that express folate receptor β (FRβ) are immunosuppressive, and CAR-T cells targeting FRβ + TAMs in the TME bring about enriched pro-inflammatory monocytes, an influx of endogenous tumor-specific CD8+ T cells, delayed tumor progression, and prolonged survival in animal models [90].

### Synergistic effects of ICB in solid tumors

Stimulatory immune checkpoint molecules can be classified into superfamilies: CD27, CD40, CD134 (OX40), and CD137 (4-1BB) belong to the TNF receptor family; CD28 and ICOS belong to the B7-CD28 superfamily. These stimulatory factors play essential roles in activating and maintaining the killing capacities of T cells [91]. In contrast, the inhibitory immune checkpoint molecules play opposite roles in suppressing of T lymphocytes. With the analysis of PD1 and CTLA-4, a new era of the treatment of ICB began. Inhibitors to CTLA-4 and PD1 were approved for melanoma patients by FDA in 2011 and 2014, respectively. Several new inhibitory immune checkpoint molecules were identified in the last decade. A high concentration of adenosine in the TME has been found, and by binding to adenosine receptors, A2AR&A2BR, activation of CTL is inhibited [92]. When it interacts with its ligand galectin-9, the T-cell immunoglobulin domain and mucin domain 3 (TIM-3) negatively regulates T cell function. Lymphocyte Activation Gene-3 (LAG-3) can suppress the immune response via Tregs and directly affect CTL. B7-H3 and B7-H4 also play an inhibitory role in stimulating T lymphocytes, while their receptors have not been identified. Indoleamine 2,3-dioxygenase (IDO) and tryptophan 2,3-dioxygenase (TDO) is tryptophan catabolic enzymes that suppress T and NK cells, and generate and activate regulatory T cells (Tregs) and myeloid-derived suppressor cells (MDSC) [93]. Sialic acid-binding immunoglobulin-type lectin (SIGLEC) is on the surface of different immune cells, including NK cells, macrophages (SIGLEC7), and activated T cells (SIGLEC9), and suppressed the immune properties by binding to terminal sialic acid on glycans on the cell surface [94, 95]. Recent advances in tumor immune checkpoint therapy have been comprehensively reviewed [89, 96, 97].

CAR-T cells express higher levels of PD1–1, TIM-3, and LAG-3. PD-L1/PD-1 can directly inactivate CD28 signaling in CAR-T cells, thereby inhibiting their function, and PD-1- or LAG-3-deficient CAR-T cells exhibit enhanced anti-tumor function in vitro and in vivo [98]. Adding PD-1 inhibitors to CD19 CAR-T therapy improved the persistence of CAR-T cells [99]. CRISPR/Cas9-mediated gene editing produced CD19 CAR-T with a LAG-3 knockout but did not increase efficacy. Applying PD-1 or TIM-3 blockade in CAR-T enhanced synergistic anti-tumor efficacy [100]. ICB and CAR-T are the two milestone achievements in cancer immunotherapy, and their combination may lead to synergistic effects. Interactions between cancer cells and components of the TME consist of an immunosuppressive condition for T lymphocytes [4]. Cancer cells can directly dampen T-cell cytotoxicity by upregulating inhibitory factors, inducting senescence, exhaustion, and apoptosis. In addition, cancer cells can recruit and re-educate various immune cells to an immunosuppressive state, such as myeloid-derived suppressor cells (MDSC), M2 tumor-associated macrophages (TAM), Tregs, N2 tumor-associated neutrophils (TAN), and regulatory B cells (Breg). These immunosuppressive cells can induce anergy of T cells by producing immunosuppressive cytokines (IL-10, TGFβ1), nutrition deprivation (arginase-1, Arg-1), inducing inhibitory co-stimulation factors, and weakening antigen-presentation [16, 101]. Some preclinical trials of ACT aimed to achieve a synergistic role in remodeling the hostile TME [102, 103].

### Outcomes of preliminary clinical trials of TCR-T treatment in solid tumors

To date, no TCR-T production has been approved by the FDA. However, during the last two decades, dozens of preliminary phase I/II studies showed promising results in solid tumors [104], especially in malignant melanoma. CSGs, such as NY-ESO-1 and MAGE, are the most popular tested targets. TSAs, including KRAS G12D, and E6/7, are also tested and show promising results [48, 49]. HLA-A*02:01-restricted NY-ESO-1-targeting-TCR-T was tested in 38 melanoma and synovial sarcoma cases, and the ORR was up to 58% [47, 105]. Although the adverse effects of TCR-T treatment were common, most were manageable [63]. The high cost, strict HLA allele restriction, and lack of specific tumor antigens limit the wide use of TCR-T. The development of ready-to-use production (allogeneic donor T-cells), high-output platforms for screening, and identification of efficient HLA-antigen-complexes will further support the progress of TCR-T treatment. The published and ongoing clinical trials of TCR-T in solid tumors are listed in Tables 1 and 2.

**Table 1:** Published clinical trials of TCR-T treatment for solid tumors

Year	Target	Type of antigen	HLA Allele	Tumor Type	NO. Patients	Efficacy (ORR, %)	Adverse Effects (%)	Clinical Trial Phase	Ref.
2022	KRASG12D	TSA	C*08:01	pancreatic carcinoma	2	50.0%	none (grade 3–5)	I	[49]
2021	E7	TSA	A*02:01	HPV-associated	12	50.0%	none (grade 3–5)	I	[72]
2021	MAGE-A4	CGA	A*02	synovial sarcoma,	37	39.0%	CRS (59.5%)	II	[63]^a^
2020	MAGE-A4	CGA	A*02	various solid tumors	5	40.0%	none (grade 3–5)	I	[63]^a^
2020	MAGE-A4	CGA	A*02	various solid tumors	34	25.0%	2 death (5.9%)	I	[63]^a^
2019	E6	TSA	A*02:01	HPV-associated solid	12	16.7%	none (grade 3–5)	I/II	[106]
2019	NY-ESO-1	CGA	A*02	synovial sarcoma	42	35.7%	CRS (11,9%)	I/II	[107]
2019	NY-ESO-1	CGA	A*02:01	various solid tumors	9	33.3%	none	I	[63]^a^
2019	NY-ESO-1	CGA	A*02:01	various solid tumors	9	22.2%	CRS (55.6%)	Ib	[63]^a^
2019	NY-ESO-1	CGA	A*02:01	various solid tumors	10	20.0%	none (grade 3–5)	I	[78]
2018	Tyrosinase	TAA	A*02	melanoma	3	33.0%	vitiligo (66.7%)	I	[108]
2018	MAGE-A10	CGA	A*02	various solid tumors	8	0.0%	CRS (25%)	I	[63]^a^
2017	MAGE-A3	CGA	DPB1*04:01	various solid tumors	17	23.5%	fever (58.8%), liver or renal dysfunction (11.8%)	I/II	[77]
2016	KRASG12D	TSA	C*08:01	colorectal carcinoma	1	partial remission	none (grade 3–5)	I	[48]
2015	NY-ESO-1	CGA	A*02:01	melanoma; synovial sarcoma	38	58.0%	none (grade 3–5)	II	[105]
2015	MAGE-A4	CGA	A*24:02	esophageal cancer	10	0.0%	none (grade 3–5)	I	[80]
2014	MART-1	TAA	A*02:01	melanoma	14	0.0%	erythematous skin rash (21.4%), acute respiratory distress syndrome (14.3%)	II	[109]
2013	MAGE-A3	CGA	A*02:01	various solid tumors	9	55.6%	neurological toxicity (33.3%)	I/II	[76]
2011	CEA	TAA	A*02:01	colorectal cancer	3	33.3%	severe colitis (100%)	I/II	[110]
2009	MART-1	TAA	A*02:01	melanoma	20	30.0%	skin toxicity (70%), uveitis (60%), hearing loss (50%)	II	[111]
2009	gp100	TAA	A*02:01	melanoma	16	18.8%	skin toxicity (93.8%), hearing loss (31.3%, uveitis (25%),	I/II	[111]
2006	MART-1	TAA	A*02:01	melanoma	17	12.0%	none	I/II	[112]

^a^Poster

**Table 2:** Registered ongoing clinical trials of TCR-T treatment for solid tumors in Clinicaltrial.org

Registration time	NO.	Status	Estimated enrollment	Target	HLA Allele	Vector	Cell sources	Tumor Type	Country
Oct-22	NCT05587543	not yet recruiting	24	EBV	NA	NA	autogenous T cells	EBV positive nasopharyngeal carcinoma	China
Oct-22	NCT05580796	not yet recruiting	50	NA	HLA-A * 02	NA	autogenous T cells	malignant solid tumors failed to receive standard treatment	China
Sep-22	NCT05539833	recruiting	50	NA	HLA-A*02	NA	autogenous T cells	various solid tumors	China
Aug-22	NCT05483491	recruiting	42	KK-LC-1	HLA-A*01:01	NA	autogenous T cells	gastric, breast, cervical, lung, and other KK-LC-1 positive cancers	China
Jun-22	NCT05438667	recruiting	11	KRAS G12V or G12D	HLA-A*11:01	NA	autogenous T cells	advanced pancreatic cancer and other solid tumors	China
Jun-22	NCT05417932	recruiting	46	HBsAg	HLA-A *02	NA	autogenous T cells	recurrent or metastatic HCC after standard systemic therapies	China
May-22	NCT05357027	recruiting	18	HPV16 E6	HLA-A*02	NA	autogenous T cells	relapsed/refractory to standard treatment or metastatic cervical carcinoma	China
Apr-22	NCT05349890	not yet recruiting	24	NA	NA	NA	autogenous T cells	metastatic or locoregionally advanced epithelial cancers	United States
Apr-22	NCT05339321	recruiting	36	HBV	HLA-A *02	NA	autogenous T cells	HBV-related HCC	China
Jan-22	NCT05194735	recruiting	180	NA	NA	Sleeping Beauty transposon/transposase system	autogenous T cells	relapsed/refractory solid tumors	United States
Jan-22	NCT05195294	not yet recruiting	55	HBV	NA	NA	autogenous T cells	advanced HBV-related HCC	United States
Nov-21	NCT05122221	recruiting	12	HPV16	NA	NA	autogenous T cells	HPV16 positive advanced cervical, anal, or head and neck cancers	China
Nov-21	NCT05124743	recruiting	2000	NA	NA	NA	autogenous T cells	various solid tumors	United States
Sep-21	NCT05035407	recruiting	100	KK-LC-1	HLA-*A01:01	NA	autogenous T cells	gastric, breast, cervical, lung and other KK-LC-1 positive epithelial cancers	United States
Mar-21	NCT04809766	recruiting	15	Mesothelin	HLA-A*02:01	NA	autogenous T cells	metastatic pancreatic ductal adenocarcinoma	United States
Feb-21	NCT04745403	recruiting	10	HBV	HLA-A*02:01 or HLA-A*24:02	NA	autogenous T cells	Hepatitis B Virus (HBV)-related HCC	China
Jan-21	NCT04729543	recruiting	20	MAGE-C2	HLA-A*02	NA	autogenous T cells	MAGE-C2-positive melanoma and head and neck cancer	Netherlands
Dec-20	NCT04677088	active, not recruiting	7	HBV	NA	NA	autogenous T cells	HCC in post liver transplantation	China
Nov-20	NCT04639245	suspended	18	MAGE-A1	HLA-A*02:01	NA	autogenous T cells	metastatic triple negative breast cancer, urothelial cancer, or non-small cell lung cancer	United States
Aug-20	NCT04509726	recruiting	20	LMBP2	NA	NA	autogenous T cells	metastatic/refractory nasopharyngeal carcinoma	China
Mar-20	NCT04318964	recruiting	12	NY-ESO-1	HLA-A * 02:01	NA	autogenous T cells	sarcoma	China
May-19	NCT03941626	recruiting	50	EGFRvIII/DR5/NY-ESO-1/Mesothelin	NA	NA	autogenous T cells	solid malignancies	China
May-19	NCT03970382	suspended	21	NA	NA	NA	autogenous T cells	locally advanced or metastatic solid tumors	United States
Mar-19	NCT03891706	recruiting	30	NA	NA	NA	NA	advanced solid tumors	China
Dec-18	NCT03778814	recruiting	30	KK-LC-1	NA	NA	autogenous T cells	lung cancer and other solid tumors	China
Nov-18	NCT03747484	recruiting	16	MCPyV	HLA-A*02	NA	autogenous T cells	metastatic or unresectable Merkel cell cancer	United States
Oct-18	NCT03691376	active, not recruiting	4	NY-ESO-1	HLA- A*02:01	NA	autogenous T cells	recurrent or treatment refractory ovarian, fallopian tube or primary peritoneal cancer	United States
Mar-18	NCT03462316	recruiting	20	NY-ESO-1	HLA-A*02:01	NA	autogenous T cells	bone and soft tissue sarcoma	China
Aug-16	NCT02858310	recruiting	180	HPV-16 E7	HLA-A*02	NA	autogenous T cells	metastatic or refractory/recurrent human papillomavirus (HPV)-16+ cancers	United States

## CAR-NK

### Mechanisms of inhibition and activation of NK cells in solid tumors

NK cells are one of the immune cells differentiated from the common lymphoid progenitor accounting for approximately 5–20% of all circulating lymphocytes in peripheral blood. Typically, NK cells are identified by CD56 expression rather than CD3 or TCR co-expression and are classified into two subpopulations depending on the CD56 and CD16 expression levels. Like most NK cells, CD56^bright^ cells are found in bone marrow, secondary lymphoid tissue, liver, and skin. By acquiring CD16, CD56^bright^ can transform into CD56^dim^. The CD56^dim^CD16^high^ NK cells are prevalent in peripheral blood, displaying powerful killing capacities. NK cells recognize and eliminate altered cells at an early stage rapidly, representing a solid innate immune surveillance to prevent tumor development and progression [113]. NK cells do not need prior sensitization, costimulatory signals, gene rearrangement, or the HLA-peptide complex. Activated NK cells eliminate altered cells via various mechanisms: releasing perforin and granzymes to form pores in target cells, leading to lysis or apoptosis; promoting FasL expression to induce apoptosis; releasing TNF-α, IFN-γ, GM-CSF, and chemokines (CCL1, CCL2, CCL3, CCL4, CCL5, and CXCL8) to recruit and activate other effector immune cells; antibody-dependent cell-mediated cytotoxicity (ADCC) by binding to the specific antibody via CD16 [114].

## Inhibitory and activating receptors of NK cells

### Killer immunoglobulin-like receptor (KIR)

Various inhibitory and activating receptors regulate the NK cell functions. They can be classified into classical or non-classical HLA class I molecules and non-HLA class I molecules according to their matched ligands [115]. Killer immunoglobulin-like receptor (KIR) and killer lectin-like receptor (KLR) are two receptor families binding classical or non-classical HLA I molecules. KIRs are a group of type I transmembrane glycoproteins and belong to the immunoglobulin superfamily (IgSF). KIRs are named after the structure regarding the number (2 or 3) of extracellular immunoglobulin-like domains (D) and the length (long or short) of the intracytoplasmic tail [116]. The extracellular domains can recognize specific HLA class I molecules. KIR2Ds interact with HLA-C allotypes and KIR3Ds interact with HLA-A/−B allotypes. The inhibitory KIRs (KIR2DL1, 2, 3, 5, and KIR3DL1–3) have long intracytoplasmic tails containing immunoreceptor tyrosine inhibitory motif (ITIM) domains phosphorylated by Src family kinases in response to interactions with their cognate HLA class I molecules, which helps recruit SHP-1 and SHP-2 phosphatases and subsequently suppress activation signals. The activated KIRs (KIR2DS1–5, KIR3DS1) have a short intracytoplasmic tail with the ITIM domain. However, they contain a positively charged lysine or arginine residue in the transmembrane domain that binds to DAP12, an adaptor molecule containing a negatively charged residue and an ITAM domain [116, 117]. KIR2DL4 is an exception to this pattern with both inhibitory and activating capacities. KIR2DL4 resides in endosomes, and its ligand is HLA-G. Soluble HLA-G accumulates in KIR2DL4 endosomes, and its activation initiates proinflammatory and proangiogenic responses through a novel endosomal signaling pathway involving serine/threonine kinases DNA-PKcs and Akt [118]. Seventeen KIR genes (including two pseudogenes) have been discovered in the leukocyte receptor complex encoded by an approximately 150-kb segment on chromosome 19q13.14 [119].

### Killer lectin-like receptor (KLR)

KLRs consist of two types II transmembrane molecules, CD94, and natural-killer group 2 (NKG2) family members, to form a heterodimer, both of which are C-type lectin family members. NKG2 family has seven members, namely NKG2A, B, C, D, E, F, and H. NKG2A and NKG2B are inhibitory receptors, and the rest are activating receptors. KLRs can also recognize non-classical HLA class I molecules (HLA-E) by the extracellular domains. HLA-E only binds a subset of peptides derived from signal peptides of classical HLA class I molecules, including HLA-A, B, C, G. Its expression on the cell surface depends on the nonamer peptide epitope derived from the signal sequence of classical HLA molecules and generated by signal peptide peptidase and the proteasome [120]. CD94 does not have an intracellular domain; however, the intracellular domain of NKG2A and NKG2B contain ITIM, inhibiting the activation of NK cells. In contrast, NKG2C, NKG2E, and NKG2H cooperate with DAP12, which contains ITAM, to activate NK cells [121].

Unlike the other members of the NKG2 family, NKG2D does not form heterodimers with CD94, and its ligand is not an HLA class I molecule. The ligands are self-induced proteins wholly absent or present at low levels on the surface of normal cells but overexpressed in infected, transformed, senescent, and stressed cells [122], regulated at transcriptional and post-transcriptional levels by diverse stress pathways [123]. One of the most prominent pathways is DNA damage response participating in NKG2D ligand upregulation. All NKG2D ligands share homology with HLA class I molecules and can be divided into two families, MIC (MHC Class I chain-related molecules) and RAET1 (Retinoic acid early transcript 1E)/ULBP (UL16 binding protein). There are seven human MIC genes (*MICA-G*), of which only *MICA* and *MICB* produce functional transcripts. Six of the ten known RAET1/ULBP genes encode functional proteins. These NKG2D ligands are expressed on the cell surface of the breast, ovarian, colorectal, cancer, and lung cancer [124, 125].

### Natural cytotoxicity receptor (NCR)

Another activating receptor of NK cells independent of HLA molecules is the natural cytotoxicity receptor (NCR) exclusively expressed in NK cells. The NCR family consists of three types I transmembrane (TM) receptors, NKp46, NKp44, and NKp30, belonging to the immunoglobulin superfamily encoded by *NCR1*, *NCR2*, and *NCR3* genes, respectively [126]. Resting and activated NK cells express NKp30 and NKp46 constitutively, while the subsequent activation induces NKp44 expression. The ζ chain CD3 signaling complex is expressed by NK cells even though they lack TCR expression. NKp30 and NKp46 promote NK cell activation by interacting with the ITAM-containing proteins CD3ζ and FcRγ. Signaling through DAP12, NKp44 mediates NK killing and IFN-γ and TNF-α releasing. Blocking individual NCRs by soluble monoclonal antibodies has limited effects on NK cell cytotoxicity, and different tumor cells respond distinctly. The combined blockade of NCRs has synergistic effects of medicating NK cell cytotoxicity in certain tumors [116]. The HLA class I molecules in healthy human cells have a higher affinity to inhibitory receptors (KIRs and KLRs), which avoid self-cytotoxicity and induce self-tolerance. However, the altered cells lose surface MHC class I expression resulting in lower inhibitory signals in NK cells. In addition, viral infection or tumor development triggers cellular stress via DNA damage response and senescence or stimulates tumor suppressor genes to upregulate activating receptors NCRs. The signals from activating receptors in NK cells shift the balance toward NK cell activation and target cell elimination, directly through NK cell-mediated cytotoxicity or indirectly through proinflammatory cytokine secretion. In the past 20 years, dozens of ligands of inhibitory and activating receptors have been discovered in cancers [127]. Several ligands of NCRs have higher expressions in cancer cells, and their soluble forms in TME are essential to induce the anergy of NK cells [128].

Heparan sulfate-glycosaminoglycans (HS-GAGs) are expressed on cell surfaces and within the extracellular membrane to form HS-proteoglycans (HSPGs). HSPGs play a vital role in tumor progression, allowing cancer cells to proliferate, progress, and metastasize. Three NCRs bind to different HS sequences, among which NKp30 and NKp46 bind to HS and NKp44 displays a different binding pattern. The interaction between cell-associated HSPG (syndecan-4) and NKp44 could adjust the membrane distribution of NKp44, thereby dampening NKp44 activity [129, 130]. NK cells are hypothesized to sense the changes in HSPGs in TME via NCRs. Aberrant regulations of key HS biosynthetic enzymes (3-O- and 6-O-Sulfotransferases), catabolic enzymes (heparinase), and the HS endosulfatases (ULF1 and SULF2) were in various tumors [131]. Silencing of heparan sulfate-glucosamine 3-O-sulfotransferase 4 (HS3ST4) in cancer cells recruits more activated NK cells in the TME [132]. Aberrant sulfation of cell-surface HSPGs can affect NK cell surveillance of tumors.

B7-H6 belongs to the B7 family of costimulatory molecules with two extracellular Ig-like domains, a membrane distal IgV domain, and a membrane-proximal IgC domain. B7-H6 interacts with NKp30 through the complementarity determining region-loops of its IgV domain, resembling the binding between antibodies and antigens. Normal cells do not express B7-H6 in contrast to high expression levels in several cancers [133, 134]. Its soluble form is produced by metalloprotease-mediated shedding B7-H6 from the cell surface. It can be detected in the serum of liver cancer, metastatic gastrointestinal tumors, neuroblastoma, and peritoneal fluid of ovarian cancer, associated with NK cell dysfunction and poor overall survival [133].

Galectin-3 is a β-galactoside-binding lectin expressed in the cytoplasm, nucleus, cell surface, or extracellularly across different cell types. The soluble galectin-3 released from tumor cells binds specifically to NKp30, inhibiting NKp30-mediated cytotoxicity [135]. HLA-B-associated transcript 3 (BAT3), also known as Bcl2-associated anthogene 6 (BAG6), is an intracellular NKp30 ligand. BAT3 can accumulate in the nucleus following DNA damage and could be released by cancer cells to involve NKp30 to activate NK cells [136]. Exosome-release BAT3 could promote the crosstalk between NK cells and dendritic cells, while plasma-soluble BAT3 suppresses NK cell activation by competing with an exosomal form of BAT3 [137].

ECM protein, Nidogen-1 (NID1), was identified as an NKp44 ligand. NID1 is an essential basement membrane component and functions in its aggregation and stabilization. When the tumor invades through the basement membrane, NID1 is shed into extracellular fluids, and the soluble NID1 can bind to NKp44 to further impair the NK cell functions to promote immune evasion [138]. The platelet-derived growth factor (PDGF) family comprises four polypeptides that can assemble into five dimeric isoforms, termed PDGF-AA, PDGF-BB, PDGF-AB, PDGF-CC, and PDGF-DD. Their activated signal pathways in tumor cells promote tumor growth, proliferation, angiogenesis, epithelial-to-mesenchymal cell transition, and metastasis. PDGF-DD is the ligand of NKp44 and can stimulate TNF-α and IFN-γ release in NK cells and contribute to cell cycle arrest in melanoma, ovarian cancer, and breast cancer by binding to NKp44. PDGF-DD can also increase NRC2 expression [139]. NKp44 interacts with proliferating nuclear antigens (PCNA). Cell surface expression of PCNA in breast cancer cells reduced NK cell cytotoxicity and IFN-γ via the NKp44–1 splice variant that potentially encodes a cytoplasmic ITIM. Preferential expression of the transcript NKp44–1 isoform encoding the ITIM domain is related to poor survival in acute myeloid leukemia [140, 141].

### Immune checkpoint receptors on NK cells

Several immune checkpoint receptors on CTLs, such as PD-1, TIGIT, CD96, TIM-3, and LAG-3, also conditionally express on NK cells. The NK cells can recognize the non-HLA class I molecules on tumor cells via these immune checkpoint receptors. The activations of these immune checkpoint receptors can negatively regulate their antitumoral functions [142–144]. PD-1-positive NK cells show a weaker antitumor function; however, blockade of the PD-1/PD-L1 axis can restore their formidable killing capacities [145]. TIGIT on NK cells can interact with CD155, CD112, and CD113. It can directly inhibit the cytotoxicity and IFN-γ production of NK cells via activation of the ITIM/ITT motif. CD96 on NK cells can also recognize CD155 on tumor cells. Although it does not inhibit the cytotoxicity of NK cells, it can lower the IFN-γ production via activation of the ITIM/YXXM motif. Higher infiltration of CD96^+^ NK cells in tumor tissue showed poorer survival in pancreatic and liver cancer patients [146]. LAG-3’s ligands on NK cells include HLA class II molecules, liver sinusoidal endothelial cell lectin (LSECtin) and fibrinogen-like protein 1 and their interaction can activate the KIEELE motif. Using IL-12 to boost the cytotoxicity of NK cells in a lung cancer murine model increased the NK cell population with higher expression of LAG-3, limiting their antimetastatic activity [147]. TIM-3 on NK cells has four main ligands, including Gal-9, phosphatidylserine, high mobility group box 1 protein (HMGB1), and carcinoembryonic antigen-related cell adhesion molecule 1(Ceacam-1). Once TIM-3 binds to its ligands, it will trigger the phosphorylation of tyrosine and release Bat3 from the cytoplasmic tail of TIM-3, leading to its inhibitory function. Tumor cells often down-regulate HLA I molecule expression as a mechanism of evasion from T cell recognition; however, it becomes more efficiently recognized and killed by NK cells. Blockades of immune checkpoint signals in NK cells have achieved synergistic roles in several tumors in murine models [148]. It should be noted that the expressions of the various ligands of immune checkpoints varied widely in different malignant tumors, and the expressions and roles of their receptors in the NK cells are inconsistent. Therefore, identifying vital immune checkpoint signal pathways of NK cells in specific tumors is the prerequisite for enhancing their tumor-killing capacities.

## Advantages of CAR-NK over CAR-T in solid tumors

### Broader cell sources

The high risk of graft versus host disease (GVHD) after transfusing allogeneic cells restricts the use of allogeneic donors of T cells for adoptive TCR-T and CAR-T treatment, while NK cells hold a shallow risk of inducing GVHD [51]. IFN-γ and TNF-α produced by activated NK cells have a much lower tendency to induce CRS and severe neurotoxicity compared to cytokines (IL-1, IL-2, IL-6, and IL-15 included) released by activated T cells. These advantages make allogeneic NK cells competent to construct CAR-NK cells for adoptive cellular immunotherapy [30]. NK cells can be directly isolated from autogenous or allogeneic peripheral blood mononuclear cells (PBMCs) from patients or healthy donors. Stimulated and expanded in NK-cell media with a cytokine cocktail, these induced-NK cells become a good manufacturing practice (GMP)-grade clinical application.

As an advanced version of LAK, cytokine-induced killer (CIK) lymphocytes are also derived from PBMCs and induced by a cytokine cocktail, including anti-CD3 antibody, IL-1α, and IL-2. It mainly consists of CD3^+^CD56^+^ and CD3^+^CD56^−^ lymphocytes, whereas the antitumor effects mainly rely on the CD3^+^CD56^+^ cells. These CIK- CD3^+^CD56^+^ cells share many advantages with NK cells, e.g., they kill in HLA independently and are endowed with a low risk of GVHD, a low tendency to induce CRS, and severe neurotoxicity. Although the clinical trials showed CIK-killing capacities in certain solid tumors, conventional CIK cellular immunotherapies have similar drawbacks to LAK [149]. Furthermore, the clinical application scale of CIK- CD3^+^CD56^+^ cells makes it a potential alternative cell origin for CAR-NK cells. Several clinical trials of CAR-CIK cellular immunotherapy for hematological malignancies and solid tumors have been reported, and substantial antitumoral effects have been demonstrated [150–152].

NK cells can also be similarly produced from umbilical cord blood (UCB). Moreover, UCB banks can provide a specific HLA type with certain NK receptor profiles [153]. One clinical trial developed by UCB revealed that none of the 11 patients treated with HLA-allogenic anti-CD19 CAR-NK developed GVHD, CRS, or neurotoxicity [26]. However, UCB-derived NK cells manifest a less mature phenotype and express a lower level of CD16, KIRs, and NCRs, and a high level of inhibitory receptors, such as NKG2A [154]. On the one hand, multiple allogeneic donors can serve as sources for one patient to meet the clinical demand with “off the shelf” products; on the other hand, these heterogenous sources make it difficult to develop a common standard for commercialization [155, 156].

NK cell lines are another available source for CAR-NK construction with an unlimited proliferative ability to quickly obtain a large quantity of NK cells. The NK-92 cell line originates from NK cell lymphoma and could be expanded in vitro by a cytokine cocktail applied in most published or ongoing CAR-NK clinical trials [30, 51]. However, lack of NKp44 and CD16, potential tumorigenesis risk, and lethal irradiation before in vivo transfusion disable NK-92 as the ideal tool for CAR-NK. Compared to PBMC-derived NK cells, CD34-positive hematopoietic stem cells (HPSCs)-derived and iPSC-derived NK cells can be more efficiently transfected by CAR-virus vectors; while these NK cells are limited clinically owing to an immature phenotype and the requirement of irradiation before in vivo transfusion [29, 157, 158]. Nevertheless, these multiple origins of NK cells provide renewable sources for the “off-the-shelf” CAR-NK products.

### CAR-NK construction

To date, most reported or ongoing trials of CAR-NK used the same CAR constructions based on the CD3ζ domain and co-stimulatory 4-1BB domain as those in CAR-T cells. CAR-NK cells exhibit substantially improved NK cell activation, cytokine production (GM-CSF and IFN-γ), and cytotoxicity [159]. It should be noted that NK cells have significantly different activation mechanisms from T cells. Therefore, exclusive CAR constructions for NK cells have been attempted based on adapter molecules, such as DAP10, DAP12, and 2B4. CD3ζ-based CAR-NK is superior to the one with DAP10, and CAR-NK containing DAP12 outperforms that with CD3ζ [160]. CAR-NK cells containing 2B4 with CD48 on target cells have a more vital tumor-killing ability, increased cytotoxicity, and more cytokine production than those with 4-1BB [161]. Ten anti-mesothelin CARs in NK-92 cells with four different transmembrane domains and intracellular signaling domains were evaluated to optimize the effects of NK cell-mediated killing. Only three CAR-NK cells contained an NKG2D TM domain and 2B4 co-stimulatory domain exhibited increased anti-tumor activity and a possible mechanism that resulted in the ability to activate signaling by recruitment of endogenous DAP10 [29].

### Dual cytotoxicity capacity

Compared to T cell-based adoptive cellular immunotherapy, CAR-NK cells have CAR-dependent and CAR-independent tumor-killing capacities, potentially more potent in killing than CAR-T or TCR-T cells. CAR-dependent killing relies on the TSAs or TAAs on the cell surface. CAR-independent killing relies on the activation of NCRs by various transmembrane or intracellular antigens. Less toxicity and lower risk of GVDH of CAR-NK provide better tolerance for the patients with adjuvant treatments, including cytotoxic chemotherapy, radiation, and molecular-targeted therapy.

### Outcomes of early and ongoing clinical trials of CAR-NK therapy in solid tumors

CAR-NK cells constructed to express CAR against TAAs such as ganglioside GD2, CD138, 2B4, and CS1 have been tested in animal models where CAR-NK cells based on the NK-92 cell line demonstrate efficient killing of tumor cells in vivo and in vitro. NKG2D-DAP10-CD3ζ-expressing CAR-NK-92 has enhanced cytotoxic activity and cytokine secretion with enhanced antitumor activity in osteosarcoma. CAR-NK-92 cells targeting wild-type and mutated EGFR displayed cytolytic activity and IFN-γ production against glioblastoma cells. CAR-NK-92 cells targeting HER2 are effective in glioblastoma tumors in a murine model [159, 162].

Dozens of clinical trials with CAR-NK are currently registered and ongoing [30, 51]. Only two trials (NCT01974479, NCT00995137) started before 2016. The most common NK cell source is the NK-92 cell line, followed by autogenous PBMCs. The majorities on ClinicalTrial.gov are CAR-NK targeting CD19, CD22, BCMA, CD33, or CD7 on hematopoietic malignancies; only few clinical trials focus on CAR-NK cells targeting solid metastatic tumors expressing TAAs, including HER2, ROBO1, PSMA, mesothelin, and MUC1, and no large-scale clinical trial in solid tumors has been reported yet [51]. In a phase I clinical trial targeting CD19-positive B-lymphoid tumors, CAR-UBC-NK cells were generated from umbilical cord blood with partially matched HLAs or without HLA matching and constructed with CD28-CD3ζ-IL15-suicidal switch (induced-inducible caspase-9). Eight of 11 patients had an objective response, including seven complete remissions without severe adverse effects [26]. An NKG2DL-targeted CAR-NK is ongoing for solid tumors (NCT03415000) based on the phenomenon that several stress molecules in tumor cells recognized by NKG2D are commonly upregulated in solid metastatic tumors [30, 51, 156]. The registered ongoing clinical trials are demonstrated in Table 3.

**Table 3:** Registered ongoing clinical trials of CAR-NK treatment for solid tumors in Clinicaltrial.org

Registration Time	NO.	Status	Estimated enrollment	Target	Intra-cellular main	Vector	Cell sources	Tumor Type	Country
**Oct-22**	NCT03692663	recruiting	9	PSMA	NA	NA	NA	metastatic castration-resistant prostate cancer	China
**Sep-22**	NCT05194709	recruiting	20	NKG2D-ligand	NA	NA	NK-92 cells	relapsed/refractory solid tumors	China
**Aug-22**	NCT05507593	recruiting	18	DLL3	NA	NA	NA	relapsed / refractory extensive stage small cell lung cancer	China
**Jun-22**	NCT05410717	recruiting	40	CLDN6	NA	NA	autologous PBMC	various solid tumors	China
**Feb-22**	NCT05248048	recruiting	9	NKG2D-ligand	NA	NA	NA	previously treated liver metastatic colorectal cancer	China
**Jan-22**	NCT05528341	recruiting	38	NKG2D-ligand	NA	NA	NA	refractory metastatic colorectal cancer	China
**Jan-22**	NCT05194709	recruiting	40	Oncofetal Trophoblast Glycoprotein (5 T4)	NA	NA	NA	advanced solid tumors	China
**Nov-21**	NCT05137275	recruiting	56	Oncofetal Trophoblast Glycoprotein (5 T4)	NA	NA	NA	locally advanced or metastatic solid tumors	China
**Dec-17**	NCT03383978	recruiting	42	HER2	CD3ζ	NA	NK-92 cells	recurrent or refractory HER2-positive glioblastoma	China

### Limitations of CAR-NK

First, similar to T cells, only a small number of NK cells infiltrate the TME. Second, NK cells have a short half-life (< 10 days), indicating the need for repeated administrations to achieve a durable response. Third, although allogeneic donors PMBC, UCB, HPSC, and iPSC are available, NK cell sources for CAR-NK, the risks of tumorigenesis, incompetence of killing, and standardization should be further considered to make “off-the-shelf” CAR-NK products.

## CAR-M

### Multiple roles of TAMs in solid tumors

Macrophages are professional phagocytes and antigen-presenting cells highly specialized in removing aging, injured, dead, and mutated cells or cell debris. Macrophages are essential in innate immunity, maintaining the communication between innate immunity and adaptive immunity, which plays a vital role in infections and tumorigenesis. There are two origins of macrophages in tumor or normal tissues: circulating monocytes in the peripheral blood and resident macrophages [4, 16].

Various receptors regulate macrophages. There are mainly two types of receptors for macrophages to recognize foreign cells or debris. Non-opsonic receptors include mannose receptor (MR), scavenger receptor (SR), and Toll-like receptor (TLR). Opsonic receptors consist of IgG Fc receptor (FcγR) and complementary receptors (CR) [163]. The components or metabolites of pathogens can be recognized by the common highly conserved pathogen-associated molecular patterns (PAMPs) through binding to non-opsonic receptors. In addition, the FcγR and CRs can enhance the phagocytosis process by targeting the Fc region against specific antigens and activating complementary systems. After activation, macrophages exert killing functions by reactive oxygen intermediates, reactive nitrogen intermediates, and lysozyme. After macrophages ingest a target cell or cellular components, the latter become trapped in phagosomes, fusing with lysosomes. Subsequently, macrophages present the antigens of the target cell or cellular components to the corresponding helper T cell by integrating antigens into HLA II molecules, indicating to effector cells that the macrophages are not a pathogen, despite having foreign antigens on their surface [164]. Plasticity and diversity are two hallmarks of macrophages. Traditionally, macrophages induced by LPS and IFN-γ in vitro are defined as “classical activation” referring to M1 polarization; macrophages induced by IL-4 and IL-13 in vitro are defined as “alternative activation type” referring to M2 polarization. M2 is further defined into M2a, M2b, M2c, and M2d. These two polarized types of macrophages have different surface or intracellular markers, cytokine spectrum, and functions [165]. The surface markers for M1 include CD80, CD86, and MHC-II; the intracellular markers include iNOS, IRF5, pSTAT1; M1 macrophages secret cytokines (IL1β, IL-6, IL-12, IL-23, and TNF-α), chemokines (CXCL9, CXLCL10, and CXCL11). The surface markers for M2 include CD163, CD206, CD204, Dectin-1, CXR1, CXCR2, and CXCR4; the intracellular markers include arg-1, IRF-4, and pSTAT6. M2 macrophages secret cytokines (IL-10, TGFβ, and IL-4), chemokines (CCL17, CCL18, CCL22, CXCL1, CXCL2, and CXCL13), growth factors (PDGF, VEGF, and EGF), matrix metalloproteinases (MMPs). M1 mainly acts pro-inflammatory to exert infection protection and anti-tumor immunity. In contrast, M2 mainly acts anti-inflammatory to exert tissue repair, angiogenesis, immunosuppression, and cancer progressions [166, 167].

TAMs in the TME can be recruited, educated, and polarized into a pro-tumoral status by a hypoxic environment, metabolites (lactic acid, succinate, itaconate, citrate, α-ketoglutarate), chemokines (CCL5, CCL7, CXCL8, and CXCL12), CSF-1, CSF-2, TGFβ, IL-4 and IL-10 produced by tumor cells and stroma cells [16]. Most TAMs in the TME have an M2-like phenotype; however, that does not imply that TAMs are equal to M2 macrophages. TAMs are stimulated by various factors in the TME, making TAMs highly heterogeneous. TAMs are the predominant immune cell subtype in the TME in pancreatic cancer, colorectal cancer, gastric cancer, non-small cell lung cancer, and melanoma. In most of these malignancies, a higher density of TAMs predicts poorer survival of the patients [15, 168]. TAMs can promote tumorigenesis, progression, metastasis, and drug resistance by inducing angiogenesis, lymph angiogenesis, promoting cancer stem cells, promoting tumor cell proliferation, promoting tumor cell migration, inducing epithelium-mesenchymal transition, inducing matrix degeneration, and inducing immunosuppression [4]. In addition, cancer cells can weaken the phagocytosis of TAMs by CD47 signaling [169, 170]. Targeting TAMs, including induction of M1 polarization, reduction of infiltration, and inhibition of M2 polarization, has promising results in murine models and preliminary clinical trials. The combination of targeting TAMs and ICB also showed synergistic roles in various solid tumor models. Interestingly, a subset of TAMs expressing folate receptor β (FRβ) possess an immunosuppressive M2-like profile, and CAR-T targeting FRβ + TAMs could selectively eliminate these TAMs and improve the CAR-T anti-mesothelin effectiveness [90]. The origin, polarization, and role of TAMs in solid tumors have been comprehensively reviewed [16, 171, 172].

### CAR-M in solid tumors

Previously, transfusing many of autologous macrophages expanded in vitro and stimulated by M1-promoting factors has been carried out in the clinical trials of solid tumors. It demonstrated the feasibility and safety of infusing up to 3 × 10^9^ autologous PBMC-derived macrophages but failed to present notable anti-tumor effects [173–175]. CAR-M has advantages in solid tumors over T cells and NK cells:

1. Relevant numbers of TAMs can extravasate from the vasculature into tumor tissues;
2. TAMs have strong phagocytosis capacities;
3. TAMs have potent antigen-presenting capacities than can further active adaptive immunity;
4. TAMs can remodel the matrix to recruit more effector lymphocytes;
5. TAMs have a longer life span (several months) with long-lasting therapeutic effects;
6. TAMs can be used as drug carriers to achieve synergistic effects with CAR-M.

### Cell sources of CAR-M

Although compared to CAR-T, TCR-T, and CAR-NK, the development of CAR-M is less mature, and less data is available; immortalized monocyte cell lines and iPSCs have been used to construct CAR-M in vitro and animal models. The majority of the available data is based on immortalized monocyte cell lines. Zhang et al. [176, 177] reported that CAR-M targeting HER2 based on the murine Raw264.7 cell line could produce more MMPs in the TME by activating the intracellular domain of CD147. Niu et al. [177] also used Raw264.7 targeting CCL19 to enhance phagocytosis capacities. Klichinsky et al. [27] found that CAR-M targeting HER2 based on the human THP-1 cell line could direct anti-tumor phagocytic activity in vitro. Morrisey et al. [178] also used the human J774A.1 cell line to construct CD19 and CD22 targeting CAR-M to obtain specific phagocytosis capacities. Zhang et al. [176] constructed CAR-M targeting CD19 with iPSCs, and these CAR-Ms displayed M1 differentiation after engaging target cells. Further, these CAR-Ms could expand and exert anti-tumor activities in vivo. Primary human macrophages can be generated from PBMCs by selecting CD14+ monocytes with the help of GM-CSF. Klichinsky et al. [27] reported that the transfusion of autologous human CAR-PBCM-derived-M targeting HER2 could prolong the survival of tumor-bearing mice and reduce the metastatic burden. Like NK cell lines, monocyte cell lines have a high risk of tumorigenesis in vivo, and lethal radiation will weaken their phagocytosis capacities. Therefore, naive monocyte cell lines are not the ideal clinical sources for CAR-M. Genetic manipulation to modify monocyte cell lines is needed to guarantee safety. Although macrophages have a low risk of GVHD, they may infiltrate various organs, including the liver, lung, skin, and others, which indicates potential serious side effects of CAR-M. Whether allogeneic PBMC-derived macrophages can be donated as a source of CAR-M is unknown due to no published evidence.

### CAR-M construction

The principles of constructing CAR for CAR-M differ from those in CAR-T and CAR-NK due to the versatile roles of macrophages. Besides cytotoxicity and phagocytosis, macrophages are located within the TME. By activation of different intracellular domains of specific signaling pathways, the role of CAR-M can be selectively augmented. CAR-Ms consisting of an scFv targeting HER2, CD19, and mesothelin, a CD8 hinge and transmembrane domain, and a CD3ζ intracellular domain are qualified for enhancing phagocytosis, inducing cytokine release, and increasing anti-tumor activity. Although CD3ζ is canonically used in CAR-T, its cytosolic domain is homologous to FcRγ that drives ADCP despite having 3 ITAM domains. CAR-Ms with CD3ζ or FcRγ activating domain function similarly in phagocytosis [179, 180]. The fibrotic environment of tumors blocks the infiltrations of various immune cells into the TME. MMPs could break down the fibrotic matrix to recruit more killer lymphocytes into the TME. Macrophages are one of the leading producers of MMPs. Therefore, Zhang et al. [176] constructed a CAR-M consisting of an extracellular scFv region targeting HER2 and an intracellular activating CD147. There, after the engagement of HER2-positive target cells, the intracellular CD147 domain was activated to trigger downstream signaling pathways to increase the production of MMPs, further induce infiltrations of T cells, and inhibit breast cancer growth in murine models. In contrast, phagocytosis, ROS production, and inflammatory cytokine secretion were unaffected. CAR-Ms could be constructed based on intracellular domains from murine phagocytic receptors, including multiple EGF-like-domains protein 10 (Megf10), FcRγ, adhesion G protein-coupled receptor B1 (Bai1) and tyrosine-protein kinase Mer (MerTK), among which only FcRγ- and Megf10-CAR-Ms exhibited antigen-specific phagocytic activities [178]. A CAR-M adopted a ligand of CCR7, CCL19, to recognize the LD^hi^CCR7^hi^ immunosuppressive cell population beyond the classical extracellular scFv region. The MerTk extracellular activation domain enables the most significant toxicity against tumor cells among MerTk, TLR2, TLR4, TLR6, and 4-1BB-CD3ζ CAR-M [177]. However, by adopting the same intracellular domain of MerTk, Morrissey et al. found that this CAR-M could not bind antigen-functionalized beads [178]. PI3k signaling enables macrophages to phagocytose large target particles, and the tandem fusion of the CD19 PI3K-recruiting domain triples the phagocytosis of complete tumor cells [178, 181].

**Fig. 4:**
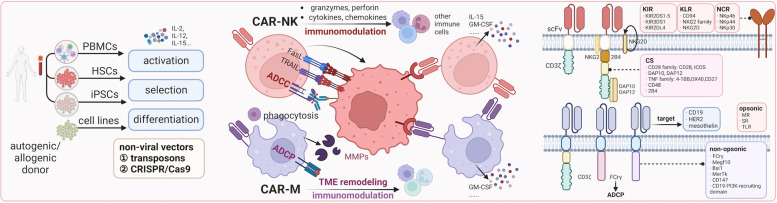
Mechanisms and development of CAR-NK and CAR-M

Macrophages defend viruses natively, so delivering any viral vectors into macrophages is technique-challenging. Novel HIV-1-derived lentiviral particles could affect myeloid cells with the viral accessory protein Vpx, which mediates the degradation of a myeloid-specific HIV-1 restriction factor (SAMHD1) to inhibit lentiviral transduction by preventing reverse transcription. As an accessible strategy for genetic manipulation, Vpx-carrying lentiviral virions can efficiently deliver transgenes to myeloid cells and accommodate any pre-existing HIV-based lentiviral vector [182, 183]. Non-integrating, replication-deficient adenoviral vectors were also explored in avoidance of the limited proliferative capacity of mature macrophages. CD46 was highly expressed in monocytes or macrophages and mediated the docking of group B adenoviruses including Ad35. Ad5f35-transduced-macrophages maintained CAR-M expression for at least 1 month in vitro and 62 days in vivo. Ad5f35 activated the macrophage inflammasome to induce M1-like CAR-Ms in solid tumors [27, 179]. Genetic modifications of macrophages can be manipulated using a variety of non-viral strategies. Deleting unmethylated cytosine-phospho-guanine (CpG) dinucleotides could help evade the detection of TLR9 and exhibit prolonged gene expression in Raw 264.7 macrophages and primary murine BMDMs. Transposon systems and CRISPR/CAS9 enable non-viral integration into the host genome and are under research in macrophages (Fig. 4) [184–187].

### Early clinical trials of CAR-M and the potential synergistic strategies in solid tumors

Clinical trials of CAR-M are still at an early stage. There is only one ongoing clinical trial (NCT04660929) based on the CAR-M, and no clinical studies of CAR-M in patients have been reported. This CAR-M is engineered with a chimeric adenoviral vector Ad5f35 carrying scFv targeting HER2 to treat HER2-positive solid tumors, still in recruitment and expected to close in February 2023 [27].

Combining CAR-M with other treatments may achieve synergistic roles. Antibody-based immunotherapies for tumors are one of the most significant advances in clinical practice. Antibodies can be designed to inhibit the activation signals in tumor cells (HER2, EGFR, CD38, RANKL), to block angiogenesis (VEGF), and to block the inhibitory signals in immune cells (CTLA4, PD1, PDL1). These antibodies can enhance ADCC and ADCP, which have formidable potential synergistic roles with CAR-Ms [188, 189]. Trastuzumab and rituximab could direct macrophages to phagocytose opsonized target cells, and antibodies blocking the “do not eat me” signal in tumor cells (CD47/SIRPα) could enhance macrophage-mediated immunotherapies [190–192]. A syngeneic CT26 model combining CAR-M with PD1 blockade improved overall survival. Antibodies can also carry chemotherapeutic drugs, which may lead to releasing TAAs and TSAs after cell lysis and could trigger immune responses [4]. CAR-M can synergize with chemotherapy or radiotherapy by inducing immunogenic cell death. Macrophages can carry various drugs into the TME by themselves, and various drugs could enhance the efficacy of CAR-Ms. [4, 193]. Macrophages can phagocytose non-particles containing chemotherapeutic drugs and genetic vectors to kill cancer cells and remodel the TME [194, 195]. Hou et al. [196] induced M1 polarized macrophages by LPS, and sorafenib-loaded lipid nanoparticles (SLNP) were incubated with M1 macrophages combining cell therapy and targeting chemotherapy.

### Limitations of CAR-M

CAR-M is still in its infancy, with no clinical trials available. There are obvious limitations of CAR-M. First, macrophages have strong anti-virus capacities, making transfection of CAR-viral vectors a tremendous technical challenge. Second, the role and functions of macrophages are dynamically changeable in the TME, which external stimuli from tumor cells and other TME components could induce. Therefore, CAR-M may display a pro-tumoral status and promote tumor progression. Mismatched HLA molecules of receipt mainly induce GVHD to donor T cells and peripheral blood pro-inflammatory cytokines, and significant CAR-cell expansion probably triggers CRS. CAR-Ms have limited expansion potential in vivo and extravasate rapidly from the peripheral blood, making GVHD and severe CRS less risky. However, the life span of macrophages can be as long as several months, which means, on the one hand, the duration of the therapeutic effects of CAR-M can last for a long time; on the other hand, potential adverse effects, such as CRS, liver failure, and neurotoxicity, may occur. Tremendous efforts are needed to develop more efficient transfection systems, control potentially life-threatening adverse effects, and avoid the disastrous pro-tumoral effects of CAR-Ms. The next generation of CARs designed and constructed by more advanced genetic manipulation may warrant further development of CAR-Ms, ultimately leading to low-cost, standardized, and “off-the-shelf” CAR-M products.

## Conclusions and perspectives

**Fig. 5:**
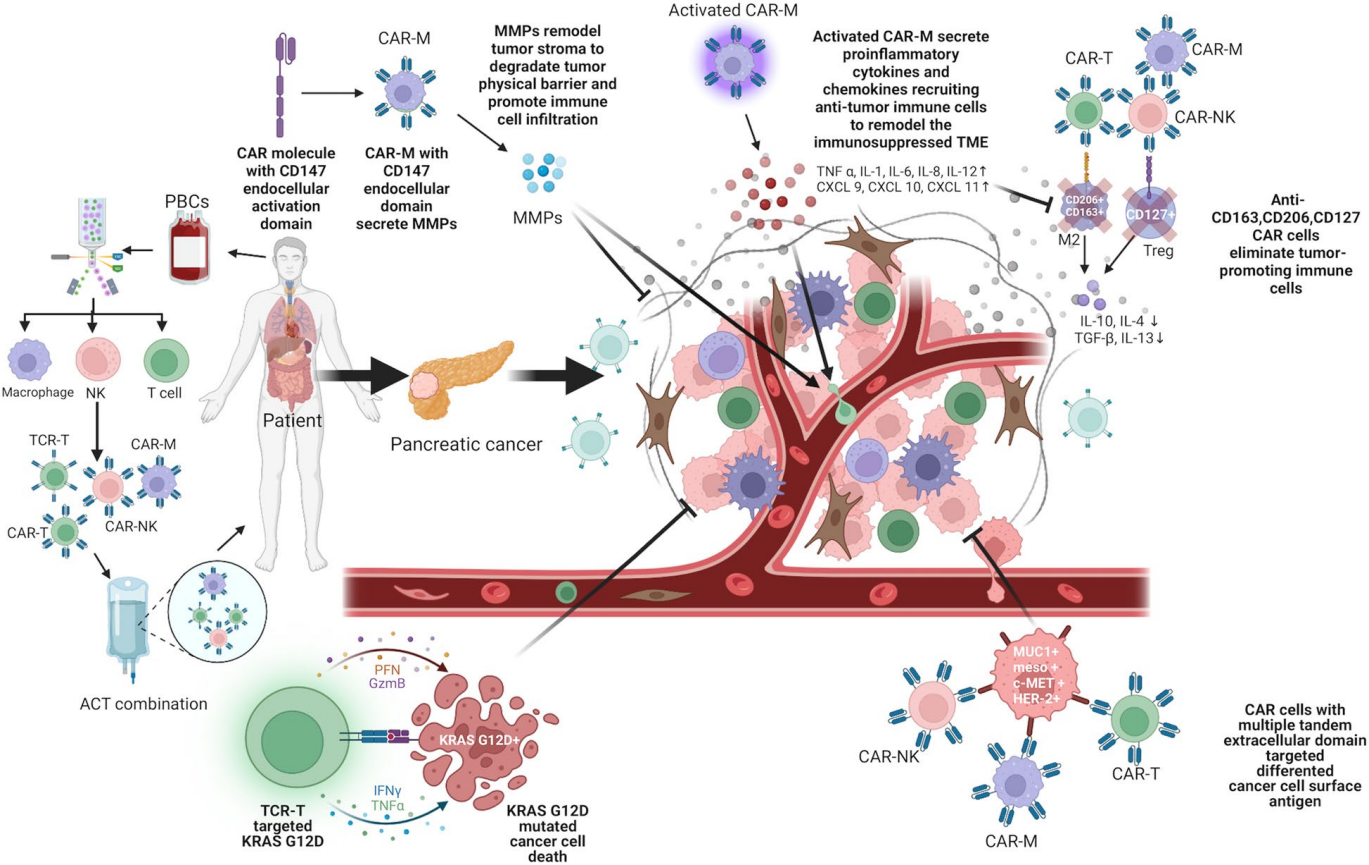
Cocktail regimen of ACTs for pancreatic cancer

During the last four decades, ACT has achieved significant clinical success in the first generation of TIL and LAK and the second generation of CAR-T. The great success of CAR-T treatment for hematopoietic malignancies is a milestone achievement in clinical immunotherapy. However, significant hurdles remain in applying ACT in solid tumors. Firstly, T cells are minor immune cell populations in TEM; And then a few solid tumors express TSAs and TAAs, which could be recognized explicitly by the CARs; Various signals from cancer cells and other stroma cells induce immunosuppression and anergy of T cells. Compared to the CAR- T cells, TCR-T, CAR-NK, and CAR-M have certain advantages as the armed force to tackle solid tumors. The comparisons of CAR-T, TCR-T, CAR-NK, and CAR-M are listed in Table 4. The ideal ACTs are assumed to be standardized and produced with comprehensive available cell sources to serve most patients in an “off-the-shelf” fashion and at an affordable cost. These engineered adoptive cells can migrate into tumors rapidly and survive for a long time to maintain long-lasting effects. They can specifically recognize, target, and kill the targeting cells by activating innate and adaptive immunity. These cells should spontaneously recognize the secondary resistance of ACTs and tackle them. Their adverse effects could be monitored and controlled. None of the current ACT alone can achieve all of these goals. The following philosophy of ACT in solid tumors could be considered in the future:

1. Combination and synergistic roles: the combinations of various types of CAR-cells (CAR-T, TCR-T, CAR-NK, and CAR-M), taking advantage of all these cells with the same or different targets, may achieve synergistic roles. For example, pancreatic cancer is an inflammatory and fibrotic tumor whose immune cells predominate in the tumor stroma [197]. M2-TAM predominates, and the fibrotic tissue blocks T cell and NK cell infiltration into the TME [15, 198]. The surface markers, mesothelin and MUC1, have higher expression in pancreatic cancer cells, and CAR-T targeting them shows a specific killing ability [199, 200]. *KRASG12D* is a relatively common mutation, and TCR-T targeting KRASG12D-HLA-C*08:02 show clinical killing ability in pancreatic cancer [49]. Therefore, combining all or some of the CAR-M-CD147, TCR-T- KRASG12D-HLA-C*08:02, CAR-T/ CAR-NK-tandem of mesothelin and MUC1, and CAR-T/CAR-NK- M2 (CD163, CD206) may achieve synergistic effects, if the side effects are well controlled (Fig. 5).

**Table 4:** Comparison of CAR-T, TCR-T, CAR-NK, and CAR-M in solid tumors

Items	CAR-T	TCR-T	CAR-NK	CAR-M
**Cell source**	autologous T cells or HLA-matched allogenic T cells	autologous T cells or HLA-matched allogenic T cells	autologous or allogenic PBMC, UC, HSC, iPSC, NK cell lines	autologous or allogenic(?) PBMC, UC(?), HSC(?), iPSC, monocyte cell lines
**Antigen**	surface antigens to CARs	intracellular antigen/HLA complex	surface antigens to CAR; ligands to KIRs, KLRs and NCRs	surface antigens to CARs; ligands to MR, SR and TLRs
**HLA restriction**	No	yes	no	no
**Intracellular activation domains**	CD3ζ /co-stimulatory domain	CD3ζ / co-stimulatory domain	CD3ζ /co-stimulatory domain, DAP10, DAP12, 2B4,	CD3ζ/ co-stimulatory domain, FcRγ, Megf10, MerTk, CD147, CD19 PI3K-recruiting domain
**Main armed force**	IL-2	IL-2	IL-15	GM-CSF
**Viral vector transfection**	high efficiency	high efficiency	moderate efficiency	low efficiency
**Intra**-**tumoral infiltration**	low	low	moderate	high
**Functions**	cytotoxicity	cytotoxicity	CAR-dependent/independent cytotoxicity, ADCC, immunomodulations	CAR-dependent/independent cytotoxicity, ADCP, immunomodulation, antigen-presenting, remodeling of TME
**GVHD risk**	high	high	low	low
**CRS risk**	high	high	low	moderate
**Other serious adverse effects risk**	high	high	low	moderate
**clinical use**	yes	no	no	no
**Ongoing clinical trials in solid tumors**	many	dozens	several	only one

**Fig. 6:**
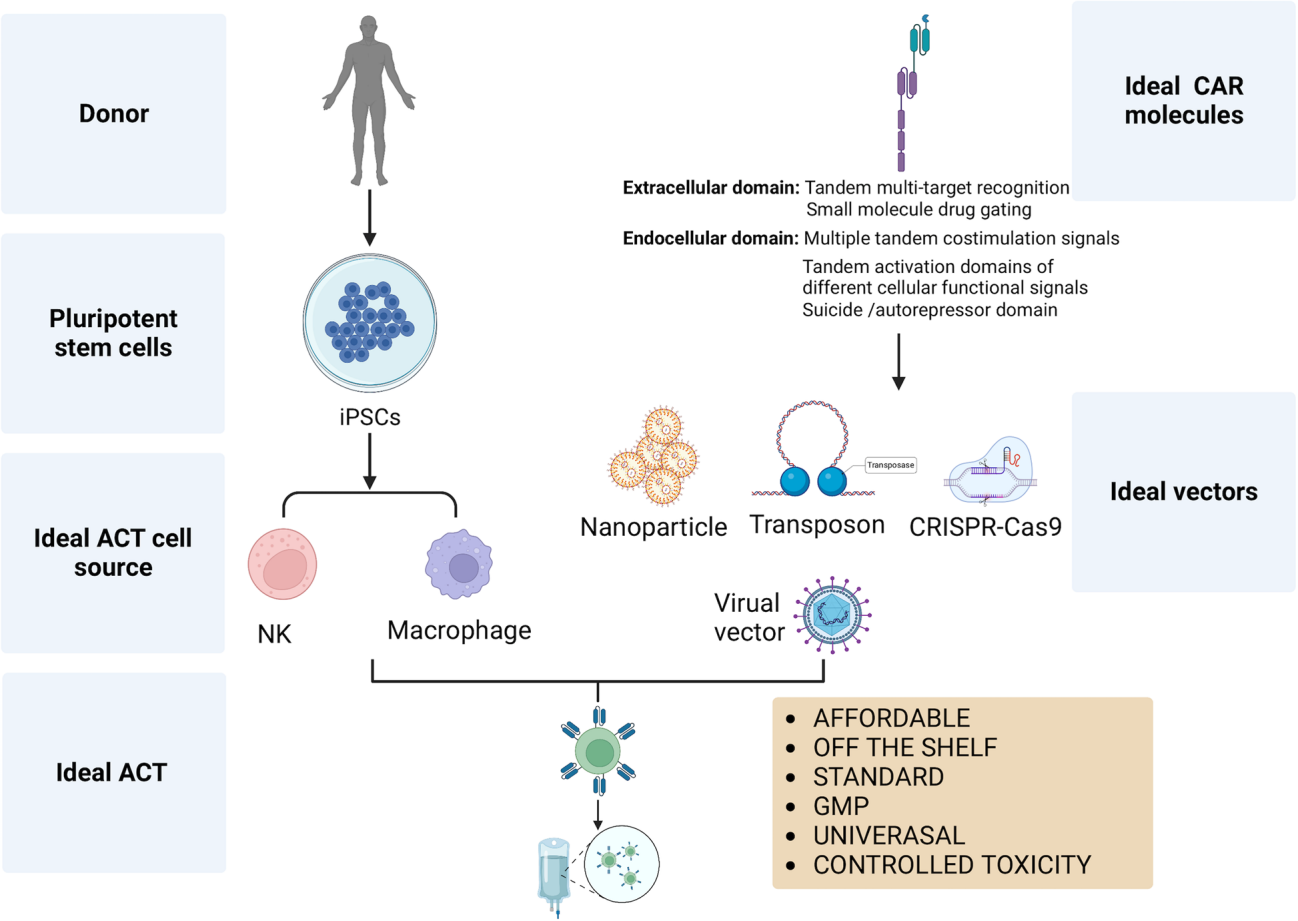
Ideal universal ACT production

(2) Design of the next generation of CARs: The ideal CARs should obtain the extracellular region that can recognize multiple TAAs or TSAs in a tandem fashion and the intracellular domains that can activate multiple tumor-killing pathways, including cytotoxicity, cytokine production, phagocytosis, matrix remodeling. The armed force domains should be designed to enhance the cells’ activation, expansion, and survival. High efficiency in the delivery of CARs in a safe and controllable manner is urgently needed. CRISPR/CAS9 and the transposon system could provide promising solutions. Recently, Susan et al. [201] reported a clinical-grade approach via CRISPR/Cas9 non-viral precision genome editing by knocking out two endogenous TCR genes and inserting neoantigen-specific TCRs in the circulating T cells. Five patients with stable disease and 11 with disease progression after standard therapy made the best response for refractory malignancies.
(3) Affordable cost, standardization, and universal production: Although several CAR-T productions for lymphoma and leukemia have been approved by the FDA and showed substantial anti-tumor effects, the price of current CAR-T is unaffordable for most patients, and it takes weeks and even months to obtain the final specific product. The iPSCs cells induced by genetic factors or chemical reagents provide practical solutions for large numbers of standardized cell sources for constructing CAR cells, which may dramatically lower costs. However, how to induce functional, competent immune cells from the iPSCs remains technique-challenging (Fig. 6).
  In conclusion, although tremendous difficulties and unpredicted hurdles remain on the road of ACT in treating solid tumors, the next generation of ACT beyond CAR-T is coming. There are great opportunities in the field of ACT for solid tumors deserving further exploration.

## Data Availability

Not applicable.

## Abbreviations

ICB: Immune checkpoint blockade
CAR-T: Chimeric antigen receptor T cell
ACT: Adoptive cell therapy
CAR-NK: CAR-natural killer cells
CAR-M: CAR-macrophage
TIL: Tumor-infiltrating lymphocyte
TCR: T cell receptor
TME: Tumor microenvironment
iPSC: Induced pluripotent stem cell
IL-2: Interleukin-2
CTL: Cytotoxic T lymphocyte
LAK: Lymphokine-activated killer cell
scFv: Single-chain fraction variable
IFN: Interferon
TNF: Tumor necrosis factor
HLA: Human leukocyte antigen
ADCC: Antibody-dependent cell-mediated cytotoxicity
NTR: Non-catalytic tyrosine-phosphorylated receptor
ITAM: Immunoreceptor tyrosine-based activation motif
CRS: Cytokine release syndrome
TSA: Tumor-specific antigen
TAA: Tumor-associated antigen
HPV: Human papillomavirus
HBV/HCV: Hepatitis B/C virus
EBV: Epstein-Barr virus
ORR: Objective response rate
CGA: Cancer germline antigen
APC: Antigen presenting cell
aAPC: Artificial antigen presenting cell
scRNAseq: Single-cell RNA sequencing
γδT cell: TCRs composed of γ- and δ-chains
αβT cell: TCRs composed of α- and β-chains
FRβ: Folate receptor β
TIM-3: T-cell immunoglobulin domain and mucin domain 3
LAG-3: Lymphocyte activation gene-3
IDO: Indoleamine 2,3-dioxygenase
TDO: Tryptophan 2,3-dioxygenase
Treg: Regulatory T cell
MDSC: Myeloid-derived suppressor cell
SIGLEC: Sialic acid-binding immunoglobulin-type lectin
TAM: M2 tumor-associated macrophage
TAN: N2 tumor-associated neutrophil
Breg: Regulatory B cell
KIR: Killer immunoglobulin-like receptor
KLR: Killer lectin-like receptor
IgSF: Immunoglobulin superfamily
ITIM: Immunoreceptor tyrosine inhibitory motif
NKG2: Natural-killer group 2
MIC: MHC Class I chain-related molecule
RAET1: Retinoic acid early transcript 1E
ULBP: UL16 binding protein
NCR: Natural cytotoxicity receptor
TM: Transmembrane
HS-CAGs: Heparan sulfate-glycosaminoglycans
HSPGs: Heparan sulfate-proteoglycans
HS3ST4: Heparan sulfate-glucosamine 3-O-sulfotransferase 4
BAT 3: HLA-B-associated transcript 3
BAG 6: Bcl2-associated anthogene 6
NID1: Nidogen-1
PDGF: Platelet-derived growth factor
PCNA: Proliferating nuclear antigen
GVHD: Graft versus host disease
PBMC: Peripheral blood mononuclear cell
GMP: Good manufacturing practice
UBC: Umbilical cord blood
HPSC: Hematopoietic stem cell
MR: Mannose receptor
SR: Scavenger receptor
TLR: Toll like receptor
FcγR: IgG Fc receptor
CR: Complementary receptor
PAMP: Pathogen associated molecular pattern
MMP: Matrix metalloproteinase
Megf10: Multiple EGF-like-domains protein 10
Bai1: Adhesion G protein-coupled receptor B1
MerTK: Tyrosine-protein kinase Mer
SAMHD1: Myeloid-specific HIV-1 restriction factor
CpG: Cytosine-phospho-guanine
SLNP: Sorafenib-loaded lipid nano-particle
